# Medical Treatment in the Home

**Published:** 1918-03-30

**Authors:** 


### A MINISTRY OF HEALTH.

### Medical Treatment in the Home.

"Preventive medicine and curative medicine are . . . in practice inseparable. Public health work will in the future . . . deal more and more with the individual, and especially with the child, both in health and in disease."—THE HOSPITAL, *September* 2, 1916.

IN our issue of February 16 we discussed the question of hospital treatment under the Poor-Law authorities. It may be well to consider briefly the present relationship of the State to medical treatment in the home.

The proposal to establish a Ministry of Health, which proposal has been pushed into prominence by various stimuli, is in reality nothing more than one for co-ordination and development—an acceptance of the need to discontinue overlapping, piecemeal legislation, and multiple administration in matters concerning the public health. The public health in this connection has already, by common consent, come to be accepted as including, with preventive medicine, a part at least of the sphere of curative treatment, both medical and surgical. The problem, therefore, is nothing less than that of propounding unified principles upon which may be founded schemes for the study, prevention, limitation and treatment of disease amongst the whole population, with the exception (in certain particulars) of the wealthier classes. Dr. Hope, medical officer of health for Liverpool, appears to fear (we think without justification) that measures for the treatment of disease may assume an importance relatively too great in comparison with measures for its prevention. "The prevention," he claims,* "and not the treatment or the cure, of disease has been most neglected in the past. . . . Treatment and alleviation always appeal to a stronger and wider sympathy than prevention; the desire to relieve in any form is one of the most amiable traits of human nature; every sentiment and every impulse are aroused to cure. Preventive measures, on the other hand, make but small appeal to the imagination; there is no sentiment about them; the more successful they are, the less apparent is the need for them. . . ."

THE PROTECTION OF THE COMMUNITY.

Whilst preventive medicine and the legal control of the practice of medicine are clearly matters for the State, it is only of recent years that the State has extended its supervision to the provision of medical treatment in the ordinary sense of the term. Under the Poor Laws treatment has long been provided for the "destitute," but the extension of this to others was an aberration from the proper functions of Poor-Law relief. Other treatment provided from public, as distinct from charitable, funds has been in the past almost entirely limited to the treatment of those who might be dangerous to the community. The principle of individual treatment has been subordinated to that of the protection of others; isolation or segregation has not only been authorised in the case of the insane, but has been a necessary precedent to the treatment in small-pox and the common infectious diseases. Submission to segregation could not be expected, still less enforced, in the absence of provision for maintenance and treatment, and these have usually been provided gratuitously. Rigorous segregation of lepers used to be enforced; they were, however, the recipients of a primitive form of poor-relief in the shape of charitable doles—the principle of the right of the indigent sick to maintenance being thus early conceded. Provision is now made in many cases from public funds for the treatment of tuberculosis; that this frequently takes the form of institutional treatment is for the good both of the patient and of the community—in fact, the practice of segregation is as far as possible encouraged in the more infectious advanced cases. The question of compulsory detention has been much discussed, especially with regard to tuberculosis and venereal disease. Vaccination is interesting as the prototype of a free prophylactic treatment, given by the State less for the benefit of the individual than for public protection.

THE FAILURE OF THE INSURANCE ACTS.

General medical treatment by the State in this country began with the treatment of school children by the education authorities: medical inspection led, naturally enough, to treatment of children for whose defects the parents were unable, or failed, to obtain treatment. The National Insurance Act introduced the principle of State-aided medical treatment for wage-earners. The co-operative method of insurance, or provision for medical care during sickness, had been previously widely adopted under the friendly societies and "clubs." It was intended that under the National Insurance Act adequate treatment should be given by general medical practitioners to insured persons, the necessary medicines and medical and surgical appliances being provided without cost to either doctor or patient. The results have not, so far, come up to expectations. Dr. Barwise, County Medical Officer of Derbyshire, says: * "The treatment given is that of the ordinary club treatment as before the Act. . . . I do not think that insured men and women have treatment in any degree better than before . . ." One mistake made in framing the provisions of the Act was the failure to recognise the present needs and conditions of medical treatment, with the resulting failure to link up the scheme with the general hospitals and other institutions and their specialists. Efficient treatment in many cases of disease or injury can nowadays be given only at or in an institution where the necessary

* *Memorandum with Constructive Suggestions on a Ministry of Health*, by Dr. E. W. Hope, Medical Officer of Health, Liverpool. 1918.

* *A Ministry of Health and a State Medical Service*, a pamphlet by Sidney Barwise, M.D. (Lond.), B.Sc., D.P.H.

**A MINISTRY OF HEALTH—(*continued*).**

facilities and skilled nursing and appliances are obtainable: the Act made no allowance for the contingency of the need for such institutional treatment, which must frequently arise. The applicability of the bottle-of-medicine form of treatment and popular faith in its all-sufficiency have both greatly diminished—the ready sale of patent medicines notwithstanding. Most of the advantages of hospital in-patient treatment are now obtainable in the better equipped of the Poor-Law hospitals or infirmaries, so that there has arisen the striking anomaly that these advantages are available for those who may lay claim to "destitution," but not to the thrifty or to those of slender means. The Local Government Committee of the Ministry of Reconstruction reports: * "It is plainly undesirable to perpetuate the existence of separate staffs, with duplicate institutions, rendering, under different Acts of Parliament, what must be to a large extent the same services whether the persons benefited be destitute or non-destitute" and points out that it should "cease to be necessary to have a duplicate provision for persons in each class of need—*i.e.*, one for those who happen to be, in the technical sense, destitute, and another for those who are not."

### Confidence in the G. P.

Domiciliary medical treatment is at present being carried out by both the district medical officer and the panel practitioner, the former paid by the Poor-Law authority from the poor-rate and the latter by the Insurance Committee from State-aided funds. In each of the two Services so provided we have a rudimentary State Medical Service. With a few trifling exceptions, neither is staffed by whole-time medical officers, and many general practitioners hold an appointment in both; consequently it may happen either that three different doctors may attend members of one household in their respective capacities of general practitioner, district medical officer, and panel practitioner, or that one doctor may so attend, combining in himself all three offices! Some degree of popular suspicion and distrust attaches to the work of both the district medical officer and the panel doctor, and even to the medicines provided upon their prescriptions: a feeling of degradation is the foundation of the dislike to being attended by the Poor-Law doctor, whilst it is by no means unusual to hear of patients who "do not go to their panel doctor" but consult another, or who even ask the panel doctor to attend them as private patients, believing that by so doing they are obtaining better attention and treatment. A whole-time Medical Service might intensify rather than dispel such objections. Although the public accepts the institution medical officer and the hospital specialist, domiciliary medical treatment is identified with the general medical practitioner, who still enjoys a high degree of reliance and confidence. Under the scheme of the National Insurance Act choice of doctor has always been strongly upheld as a desideratum. The Ministry of Reconstruction Committee has clearly in mind the need for a continuance of the work now done by Poor-Law district medical officers; the Report recommends that "medical assistance in the home be given by the staff of the County or Borough Medical Officer of Health under Public Health Acts suitably extended." There is in this recommendation a definite suggestion, if nothing more than a suggestion, of a whole-time State Medical Service.

### Subsidies to Hospitals.

It is seldom that any links exist between the work of either the district medical officer or the panel practitioner and that of the voluntarily maintained general hospitals except such as have been provided by the hospital authorities and the doctors themselves for mutual convenience and the public good: official co-ordination is wanting. There can be no doubt that the great hospital system of the country must be profoundly influenced by such an expansion of State Medical Services as would follow upon the formation of a Health Ministry. The system must be harmonised, and its unique facilities for treatment and medical education must be made available for the special treatment of all classes of the community. The trend of recent events seems to show that development is likely to proceed on the lines of the payment of subsidies to hospitals for work done. Already large sums are being thus paid from public funds: institutions for the treatment of the tuberculous, which until a few years ago were supported by subscriptions, are now mainly dependent upon payments made by county councils and insurance committees, whilst, during the past year, the London hospitals at which venereal diseases are treated have been subsidised for such treatment.

* *Report on Transfer of Functions of Poor-law Authorities in England and Wales.* 1918.

